# Optimising diagnosis and management of kidney disease: an implementation trial of a clinical decision support system future health today

**DOI:** 10.1186/s12882-024-03489-y

**Published:** 2024-02-16

**Authors:** Hannah Wallace, Qiumian Wang, Tanita Botha, Barbara Hunter, Natalie Lumsden, Craig Nelson

**Affiliations:** 1 Department of Medicine, Faulty of Medicine, Dentistry and Health Sciences, Western Clinical School, The University of Melbourne, Melbourne, VIC Australia; 2 Western Health Chronic Disease Alliance, Western Health, Footscray, VIC Australia; 3 Biostatistics Units, Faculty of Health, Deakin University, Geelong, VIC Australia; 4 Department of General Practice and Primary Care, Faulty of Medicine, Dentistry and Health Sciences, The University of Melbourne, Melbourne, VIC Australia

**Keywords:** Chronic kidney disease, Primary care, Clinical decision support software, Quality improvement, Implementation trial

## Abstract

**Background:**

Chronic kidney disease affects more than 10% of the world’s population and is a non-communicable disease of global concern and priority. There is a significant implementation gap between best practice guideline recommendations and current kidney disease management. Previous research has shown the need to partner with primary care to improve education, collaboration, and kidney disease awareness. This implementation trial will explore use of an innovative clinical decision support software, Future Health Today, to improve screening, diagnosis, and management of kidney disease in primary care. The program will be supported by tertiary care outreach services. The primary aim is to test the hypothesis that the Future Health Today implementation program will improve screening, diagnosis, and management of kidney disease. Secondary aims are to evaluate primary care satisfaction and broader health service impacts.

**Methods:**

This pre-post implementation trial using an interrupted time series design will evaluate the clinical and service outcomes of Future Health Today, using a mixed methods study in twenty general practices with an estimated population size of 150,000. Deidentified patient data will be extracted from participating practices to examine the primary aims of the study. Surveys and semi-structured interviews with general practice will inform secondary hypotheses. Data linkage between primary care and tertiary care data will examine the broader health service impacts.

**Discussion:**

This investigator driven trial will assess the impact of Future Health Today software coupled with education and clinical outreach support. Investigators hypothesise that there will be improvement in appropriate screening, diagnosis, and management of kidney disease. This program has the potential to be scaled more broadly.

**Trial Registration:**

Australian New Zealand Clinical Trial Registry: ACTRN12623001096640.

**Supplementary Information:**

The online version contains supplementary material available at 10.1186/s12882-024-03489-y.

## Background

Chronic kidney disease (CKD) is estimated to affect more than 10% of the world’s population [1, 2]. There has been no improvement in global age standardised mortality rate from 1990 to 2017, despite significant improvements in other chronic conditions such as cardiovascular disease, cancer, and chronic obstructive pulmonary disease [3, 4]. Without fundamental improvements in care, CKD is estimated to be the 5th leading cause of years of life lost by 2040 [5].

A significant proportion of CKD is preventable owing to modifiable risk factors. Earlier detection and management can change the course of disease. The majority of CKD in Australia is diagnosed and managed in primary care, making this an important focus for quality improvement. A systematic review by Neale et al. identified common themes that were barriers to detection and management of CKD in primary care including time constraints, lack of knowledge or anxiety in providing a CKD diagnosis, and difficulty interpreting CKD guidelines [6]. They found technology and a collaborative approach were important enablers of improvement [6].

There has been significant work in Australia to utilize electronic clinical decision support systems to improve detection and management of CKD in primary care, in tandem with primary care education and improved collaboration with specialist services, however previous trials were limited by intensive resourcing, workflow integration and scalability [7, 8].

Future Health Today (FHT) is a clinical decision support and quality improvement software that has been developed by co-design with primary care physicians, practice nurses, hospital physicians and technology specialists, with the development and initial pilot previously published [9–11]. This trial aims to assess the clinical outcomes, clinician satisfaction and health services impacts of this new model of care incorporating FHT, education and outreach support.

### Ethics

Ethics approval for the Future Health Today implementation trial has been obtained from the Science, Technology, Engineering, Mathematics and Medicine ethics committee at the University of Melbourne 2023-27529-44500-3. Consent will be obtained at the level of the primary care practice. The ethics committee have approved a waiver of consent for the use of non-re-identifiable routinely collected patient data. Patients can opt-out of data collection by informing practice staff. The trial is registered with the Australia and New Zealand Clinical Trials Register (ACTRN12623001096640).

## Methods

### Study design

This will be a pragmatic implementation trial using an interruptive time series design comparing outcomes in the three years prior to trial commencement with three years of active trial. Primary care data will be linked with tertiary care administrative data to enable analysis of broader health service impact. The overall program evaluation will use a mixed methods approach using the clinical performance feedback intervention theory framework [12].

### Study population

This study will take place in twenty general practices in western Melbourne and will be supported by the local tertiary hospital. This is an area with high burden of chronic disease.

#### Inclusion criteria

Primary care practices in the western Melbourne catchment and their adult patients will be eligible for inclusion.

#### Exclusion criteria

Sites without the technical infrastructure to support the Future Health Today platform (the platform works with commonly used practice software Best Practice and Medical Director). Patients identified as having a renal transplant or dialysis will be excluded from the primary analysis.

#### Recruitment

 Recruitment will take place at the practice level and will focus on practices who have been identified to serve a patient population with a significant burden of chronic disease with complex care needs. These practices have been identified through a hospital program to support patients with high risk of hospital re-admission in the context of chronic disease. Recruitment will then be expanded to the broader tertiary centre catchment area as required.

### Intervention

**Fig. 1:**
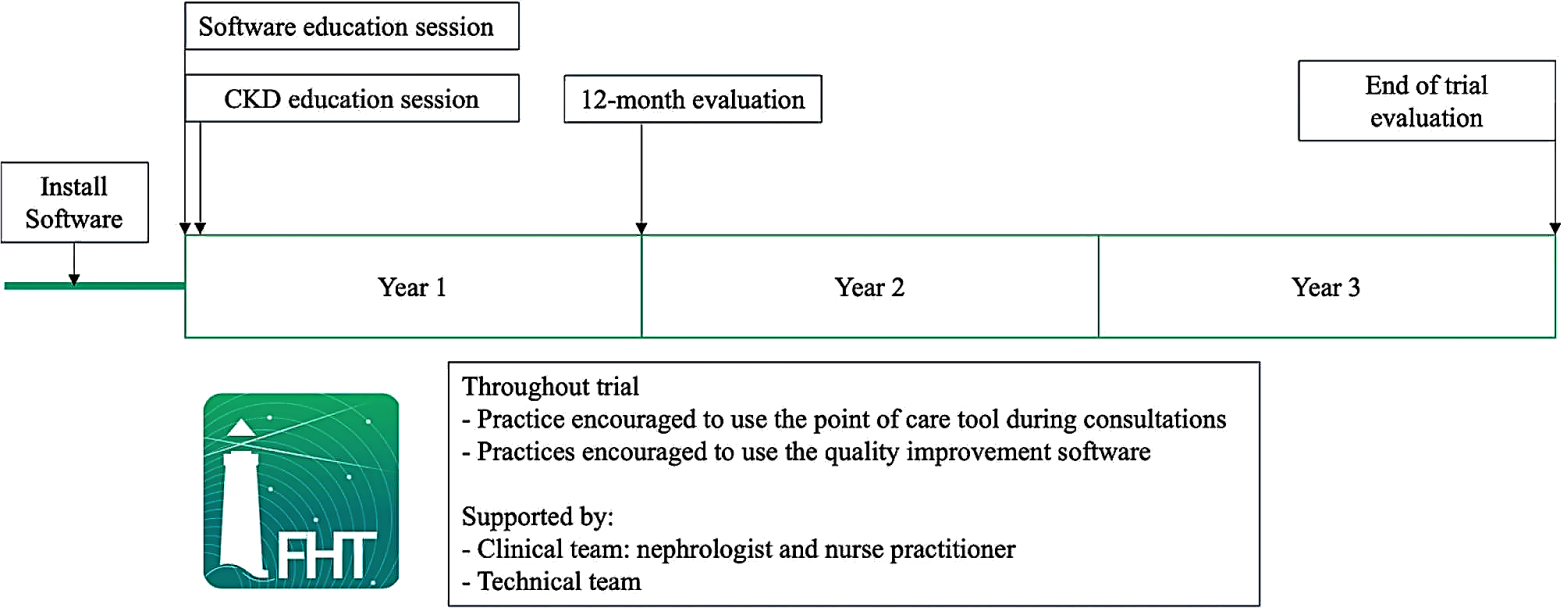
Summary of intervention future health today implementation trial

A summary of the intervention is presented in Fig. 1. FHT software with kidney disease module and related cardiovascular disease and diabetes modules will be installed on primary care computers.

Sites will be offered a software education session and provided with training videos (Supplemental appendix 1). Practices will also be provided a CKD education session (Supplemental appendix 2) and ongoing support by a nephrologist and nephrology nurse practitioner as required. During the trial practices will be encouraged to use the cohort function and complete quality improvement activities, commencing with screening patients at risk and providing a diagnosis to those with biochemical signs of CKD. Trial staff will assist practices by creating these initial cohorts.

Sites will be asked to nominate a practice champion. Practice champions will be a point of contact for the duration of the trial and asked to promote use of FHT at their practice site. They will also be providing survey and interview feedback at the end of year one and three, in addition to at least one primary care physician at the practice (Supplemental appendix 3–5).

Primary care data will be linked with tertiary centre records at baseline and then 6 monthly to end of the trial. Analysis of the retrospective cohort will be undertaken at baseline, with interim analyses yearly to end of trial. Yearly interim analysis will enable any program issues to be identified and addressed and identify areas where primary care practices may require further support. Evaluation of usability, feasibility and satisfaction will be undertaken at the end of year one and at the end of the trial.

### Outcomes

Primary outcome: change in the number of patients who are meeting quality of care indicators in three domains:

1. Screening: an appropriately timed kidney health check in patients at risk of kidney disease.
2. Diagnosis: total number of patients with coded diagnosis of CKD.
3. Management: patients with CKD on a renin angiotensin system inhibitors with at least 12 months follow up following a CKD diagnosis.

Secondary outcomes: these are divided into patient centric, health centric and overall evaluation.

Patient centric secondary outcomes:

1.1 Change in number of patients achieving the kidney cycle of care. This involves monitoring of renal function, albuminuria, diabetes (if diabetic), fasting lipids and blood pressure measurement.
1.2 Change in proportion of patients on any antiproteinuric medication including angiotensin converting enzyme inhibitor (ACE inhibitor) or angiotensin receptor blocker (ARB), sodium glucose co-transporter 2 inhibitor (SGLT2 inhibitor) and mineralocorticoid antagonists (MRA). With sub analysis of each medication class.
1.3 Change in proportion of patients with type two diabetes and CKD prescribed an SGLT2 inhibitor.
1.4 Change in urinary albumin to creatinine ratio (uACR) in patients with CKD diagnosis and at least 12 months follow up.
1.5 Change in slope of estimated glomerular filtration rate (eGFR) in patients with a coded diagnosis of CKD compared 2 years prior and 2 years post and adjusted for baseline function. The index date will be CKD diagnosis coded or if CKD already diagnosed at entry into study.
1.6 Subgroup analysis: Subgroup analysis will be undertaken comparing those with a coded diagnosis of CKD, to those with measured CKD (all patients meeting CKD diagnostic criteria) and possible CKD (patients who have one eGFR or uACR in diagnostic criteria). This will be conducted as CKD is well known to be under coded in the medical health record. CKD will be defined according to the Kidney Disease Improving Global Outcomes criteria [13].
1.7 Potential further subgroup analysis will be conducted with frequent, moderate, and minimal users of FHT (if a difference is found between practices).

Health centric secondary outcomes:

2.1 Change in referral patterns to tertiary services from practices participating in FHT.
2.2 Change in pattern of late referrals to nephrology services. This will be benchmarked against the National average and tertiary centre average.
2.3 Change in number of admissions to the tertiary centre with CKD being newly diagnosed in participating practices.

#### Overall project evaluation

A mixed methods approach with survey feedback and qualitative interview will be conducted to understand primary care satisfaction with the software including usability, feasibility, and implementation.

### Data collection and management

Three years of historical primary practice data will be collected (historical control) and compared with three years of active intervention. Primary practice data will be extracted from the primary care electronic medical record and FHT database in a non-re-identifiable format using software Grhanite™ [14]. Grhanite™ software enables data linkage without needing re-identification of patient data [14]. FHT usage will be extracted from the FHT monitoring system. Hospital utilisation, including admission and outpatient attendance to relevant specialties will be extracted from the hospital electronic record system. Hospital data will be converted to a non-re-identifiable format using Grhanite™ prior to being provided to the research team and then subsequently linked to the primary care data. All data will be housed on a secure server. Survey data will be collected using REDCap electronic survey and data capture tools hosted by Western Health [15, 16]. REDCap is a secure web-based software platform designed to support data capture for research studies [15, 16]. Practice interviews will be recorded and transcribed using a voice-to-text function, and then manually checked for accuracy.

### Data analysis

Descriptive statistics will be used to describe the baseline characteristics in the historical control and active group. Primary end points will be calculated, and a chi square test will be used to assess if the difference between pre and post-test is significant for the primary outcomes (nominal data), with *p* < 0.05 considered significant.

Univariate analysis will be undertaken for each of the outcomes to compare differences in the characteristics of the historical control versus the three year active intervention. For continuous variables, the data will be tested for normality using Shapiro Wilk test and equality of variances using Levene’s test. If these assumptions hold true, parametric tests like the t-test or ANOVA will be used. If these assumptions do not hold then nonparametric alternatives will be considered. For categorical variables a Chi Squared test will be used. When comparing multiple groups, post hoc investigations may be included with Bonferroni corrections. Regression models will be used to control for co-variates.

The analysis will be undertaken 3 years prior to study commencement (historical control) and then interim analysis yearly until trial conclusion.

Survey responses will be analysed to understand the usability of FHT software and if practice staff felt the software aided their management of CKD. Semi structured interviews of practice champions and additional primary care physicians will be conducted via zoom or phone, according to interviewees preference. Interview transcripts will be coded by at least two researchers. The clinical performance feedback intervention theory framework will be used for the overall implementation evaluation combining survey and interview data [12].

## Discussion

This study describes an evaluation of a real-world implementation trial of a clinical decision support and quality improvement software for CKD in the primary care setting, supported by the tertiary health service. This is in contrast to the traditional silos that exist between the primary and tertiary healthcare system in Australia. The trial design enables evaluation of clinical and health service outcomes of the intervention, in addition to an implementation evaluation.

There has been considerable effort to improve chronic disease management in primary care and a systematic review by Neale et al. identified that technology and enhanced collaboration were important enablers in improving CKD care in primary care [6]. There has been mixed success with clinical decision support software (CDSS) more broadly with high heterogeneity between interventions [17, 18]. A qualitative systematic review identified key recommendations for CDSS implementation including enhancing the clinical process, providing relevant clinical information, appropriate education and training, integration into standard workflow, and need for further research and design in multimorbidity [19]. This trial aims to address these recommendations with the co-design process ensuring integration into current primary care workflows, staff education and ongoing support, and use of a practice champion. Additionally, whilst the focus of this protocol is analysis of kidney disease outcomes, included modules in the FHT program address the common co-morbidities of diabetes and cardiovascular disease.

Earlier detection and management of CKD requires time for improvements to be realised, hence a three-year trial duration was chosen. One limitation of a pre-post implementation trial with a historical control is the inability to assess direct causation. This approach was chosen given the practical challenges of a randomised trial in primary care over three years in addition to the ethical challenges of preventing practices from undertaking quality improvement and CKD educational activities over this extended duration. To help address this limitation an interrupted time series design has been chosen with a 3-year retrospective control compared to the 3-year prospective intervention used to assess the rate of change in parameters over time. This will assist in determining if improvements are due to a natural increase in screening, diagnosis, and management over the course of time, or associated with the intervention.

### Electronic supplementary material

Below is the link to the electronic supplementary material.

**Supplementary Material 1:** Education outline, clinician surveys and interview questions

## Data Availability

Data sharing is not applicable to this article as no datasets were generated or analysed during protocol development. An educational outline, survey and interview templates are in the supplemental appendix.

## Abbreviations

ACE inhibitor: angiotensin converting enzyme inhibitor
ARB: angiotensin receptor blocker
CKD: chronic kidney disease
eGFR: estimated glomerular filtration rate
FHT: Future Health Today
MRA: mineralocorticoid antagonist
SGLT2 inhibitor: sodium glucose co–transporter 2 inhibitor
uACR: urinary albumin to creatinine ratio
