# Supplementary material for: Optimising diagnosis and management of kidney disease: an implementation trial of a clinical decision support system future health today
Source: BMC Nephrol. 2024 Feb 16;25:57. doi: 10.1186/s12882-024-03489-y (PMC10870544; doi:10.1186/s12882-024-03489-y)
Supplement: Supplementary file 1 — Supplementary Material 1: Education outline, clinician surveys and interview questions [file 12882_2024_3489_MOESM1_ESM.docx]

**Supplemental Appendix:**

**Supplemental Appendix 1: Technical Education Outline**

**Supplemental Appendix 2: Clinical Education Outline**

**Supplemental Appendix 3: Survey Questions Future Health Today Staff Evaluation**

**Supplemental Appendix 4: Survey Questions regarding Education Sessions**

**Supplemental Appendix 5 Interview evaluation**

**Supplemental Appendix 1: Technical Education Outline**

Sessions will be either offered via teleconference with technical team (up to 45 mins) or if preferred recorded videos can be access by staff at a convenient time.

Teleconference Education

1. What is FHT
2. Why use FHT
3. The point of care tool
4. The FHT portal homepage and the cohort tool
5. Questions

Recorded Videos:

- How to use the FHT cohort tool to [Review cohort](https://www.youtube.com/watch?v=hi3uULETDwU)
- How to use the FHT  clinical decision support tool - [Future Health Today Point of Care Training](https://www.youtube.com/watch?v=V_hv4dKMQkE)

User guides

1. FHT User Guide
2. FHT Quick Guide
3. FHT Tips and Troubleshooting Guide

**Supplemental Appendix 2: Clinical Education Outline**

Sessions can be delivered in a multiple short session format or a comprehensive education session.

- 1. Kidney Disease: Why are we worried?
  2. Epidemiology of kidney disease – Australian and Local Trends
  3. Complications of kidney disease

1. Who is at risk for kidney disease?

2.1 Risk factors for kidney disease

2.2 How to screen for kidney disease

1. Management of kidney disease

3.1 Lifestyle: smoking cessation, physical activity, weigh reduction if overweight, salt reduction

3.2 New blood pressure targets

3.3 Cardiovascular risk management

3.4 Medication management

- Angiotensin converting enzyme inhibitors or angiotensin receptor blockers

- Sodium glucose 2 co-transporter inhibitors

- Mineralocorticoid receptor antagonists

- *Glucagon-like Peptide-1 receptor agonists* (currently Australian pharmaceutical listing for diabetes)

For each medication indication, contraindications, mechanism of action, comorbidity benefits, monitoring, key practice points and patient education will be covered.

1. When to refer?

4.1 Kidney Health Australia and local referral guidelines

4.2 Pathways for referral

1. Case studies and clinical conundrums

5.1 Review of cases – general practitioners will be invited to submit cases (alternatively education team has several common cases)

5.2 Clinical questions

**Supplemental Appendix 3: Survey Questions Future Health Today Staff Evaluation**

Gender:

- Male
- Female
- Another term

Age:

- 20-34
- 35-49
- 50-64
- 65 and over

Are you a:

- GP
- Practice nurse
- Practice manager
- Other ___________

How long have you been working in this role (years)?

Clinic billing structure

- Bulk billing
- Mixed billing
- Private billing only

Electronic medical record used

- Best Practice
- Medical Directory

Other than Future Health Today, do you use any systems for quality improvement, audit or clinical decision support? (please tick all that apply)

- PenCAT
- POLAR
- cdmNET
- Canning Tool (improvement foundation)
- NPS MedicineInsight Portal
- Doctors Control Panel
- Other

Have you needed access or help to support use of FHT?

Who has provided help or support? (please tick all that apply)

- Practice champion
- Colleague
- FHT Project team
- Written guide
- Online videos

Would you recommend FHT to others?

- Yes
- No
- Uncertain

Please describe why / why not?

How long have you been using FHT?

- 1 month
- 2-6 months
- 6-12 months
- 12-24 months
- 24-36 months
- >36 months

Which components have you used? Please tick all that apply

- Point of care
- Cohort builder
- Cohort review
- Approval
- Recall
- Defer
- Quality improvement
- Education
- Reporting
- Guidelines
- Training videos
- Other resources

System usability scale. Please indicate the extent to which you agree or disagree with the following statements. (1 = strongly disagree, 5 = strongly agree)

- I think I would like to use this system frequently
- I found the system unnecessarily complex
- I thought the system was easy to use
- I think that I would need the use of a technical person to be able to use the system
- I found the various functions in the system were well integrated
- I thought there was too much inconsistency in this system
- I would imagine that people would learn to use this system very quickly
- I found this system very cumbersome to use
- I felt very confident using the system
- I needed to learn a lot of things before I could get going with this system

Do you have any other feedback for us on Future Health Today?

I have used the point of care tool for chronic kidney disease or diabetes

- Yes
- No

This is referring to the "pop up" recommendation on a patient's file and used by a General Practitioner in the consultation. If you are not a GP please tick no.

The next section is only for GPs in regards to the point of care tool.

Diagnosis and management of chronic kidney disease
Please indicate the extent to which you agree or disagree with the following statements. (1 = strongly disagree, 5 = strongly agree)
*Please note the tool is referring to the point of care "pop up" recommendations.*

- The chronic kidney disease tool helped me diagnose patients with CKD
- The chronic kidney disease tool was easy to understand
- The recommendations in the chronic kidney disease tool were relevant to my patients with CKD
- The chronic kidney disease tool helped me prescribe appropriate medications for CKD
- The links to Kidney Health Australia guidelines is helpful
- The links to kidney disease patient information is helpful
- The chronic kidney disease tool improved my practice in managing patients with CKD
- The related cardiovascular and diabetes tool were useful in managing my patients

Did you have any further feedback about the point of care tools in Future Health today? (free text response)

**Supplemental Appendix 4: Survey Questions regarding Education Sessions**

Gender:

- Male
- Female
- Another term

Age:

- 20-34
- 35-49
- 50-64
- 65 and over

Are you a:

- GP
- Practice nurse
- Practice manager
- Other

Today’s session was delivered:

- Face to face
- Zoom / online
- Recording

Feedback on the chronic kidney disease education session:
Please indicate the extent to which you agree or disagree with the following statements (1 = strongly disagree, 5 = strongly agree)

- Today’s session was informative
- The presenters were engaging
- My questions about chronic kidney disease were answered
- My questions about Future Health Today were answered
- My knowledge of chronic kidney disease has improved as a result of todays session
- I feel supported to manage my patients with chronic kidney disease
- The session was good use of my time

Did you have any feedback for the presenters or research team? (free text)

**Supplemental Appendix 5 Interview evaluation**

Semi structured interview

I am talking to you because you’ve been nominated as a user of Future Health Today.

I have a few brief background questions if you feel comfortable:

- What is your gender identity?
- Which age bracket best describes you?
  - Aged 20-34
  - Aged 35-49
  - Aged 50-64
  - Aged 65 and over
- What is your role in the clinic?

Can you describe how you have been using Future Health Today?

- - *When do you use it?*
  - *How do you use it (e.g., in the PoC, do you click on anything?)*
  - *How long does it take to read and respond to the recommendations?*
  - *Have you accessed the portal and cohort tool? If yes, probe when/what for?*

If not using FHT point of care tool: What are the reasons behind not using FHT?

- *Explore potential reasons:*
- *Do you trust the recommendations in the FHT algorithm?*
- *Does it identify patients appropriately?*
- *Do you feel it is clinically relevant?*
- *Does the reason for the patient appointment affect if you will use the software?*

Do you think Future Health Today has helped you identify and manage patients with kidney disease? Why do you think that is?

- *How useful are the recommendations for identifying chronic kidney disease?*
- *Is FHT flagging patients who would not have been followed up otherwise?*
- *How useful are the recommendation for managing patients with chronic kidney disease?*
- *Do you trust the recommendations provided by the software?*

Has the Future Health Today program changed your understanding of kidney disease?

- *How has it changed?*

In relation to diabetes, do you think Future Health Today has helped you identify and manage patients with diabetes? Why do you think that is?

- *How useful are the recommendations for identifying patients with diabetes?*
- *Is FHT flagging patients who would not have been followed up otherwise?*
- *How useful are the recommendation for managing patients with diabetes?*
- *Do you trust the recommendations provided by the software?*

Has Future Health Today program changed you understanding of diabetes?

- *How has it changed?*

Do you or your practice use the cohort tool?

If not: are there any reasons why it is not being used?

If using:

- *How do you use it?*
- *Have you used it to recall patients?*
- *What is working well or not working well? Do staff in the practice use the FHT tool?*
- *Have patients been receptive to being recalled?*

Have there been any other outcomes or unexpected outcomes of Future Health Today Chronic disease modules?

How has Future Health changed your workflow?

- *When do you look at the FHT point of care tool during the consultation?*
- *When in the consultation do you deal with the recommendation from the FHT tool?*
- *How does it affect the flow of the consultation?*
- *How often would you defer a recommendation? What are the reasons for deferring or not actioning a recommendation?*

If available, would you continue to use Future Health Today Chronic disease modules after the study?

- *Why / why not?*
- *Do you think your practice would consider paying of the software at the end of the three-year trial? Why / Why not?*

Is there anything we do to can improve the Future Health Today platform?

The second part of this interview is to assess relationship between primary care and tertiary services in relation to kidney disease and diabetes. The following questions relate to this:

What is your experience with the hospital nephrology team including referrals, communication, specialist, and nurse practitioner?

- *Are your patients being seen in a timely manner?*
- *Do you know who you could call if you had an urgent kidney concern?*
- *If you have referred a patient for review, do you receive communication from the specialist?*
- *How often is the communication from the specialist relevant?*
- *If a patient is discharged from the kidney service, do you feel like there is clear advice re ongoing management suggestions and when to re-refer?*

Has your experience (re: hospital nephrology interaction) changed during the Future Health Today study?

Were you aware of the new renal CKD nurse practitioner role? If yes, how have you found this service?

Is there anything that you would like to see change with the interaction between primary care and nephrology services?

Similarly, is there any feedback in relation the hospital diabetes service?
